# Supplementary material for: A Systematic Review of Clinical Practice Guidelines on the Management of Malnutrition in Children with Congenital Heart Disease
Source: Nutrients. 2024 Aug 20;16(16):2778. doi: 10.3390/nu16162778 (PMC11356818; doi:10.3390/nu16162778)
Supplement: Supplementary file 1 [file nutrients-16-02778-s001.zip › nutrients-3161536-supplementary.pdf]

SUPPLEMENTARY MATERIALS

Table S1. Search strategy

**#44**	**#32 AND #43**	**345**
**#43**	**#33 OR #34 OR #35 OR #36 OR #37 OR #38 OR #39 OR #40 OR #41**	**1594646**
**#41**	**cpg:ti,ab OR cpgs:ti,ab**	**49405**
**#40**	**'expert opinion':ti,ab OR 'expert consensus':ti,ab**	**41873**
**#39**	**((clinical* OR practi*) NEAR/2 (guideline* OR guidance OR protocol* OR path* OR consensus OR recommendation* OR statement*)):ti,ab**	**253357**
**#38**	**'good clinical practice':ti,ab**	**3356**
**#37**	**'consensus development':ti,ab OR 'consensus statement':ti,ab OR 'consensus statements':ti,ab OR 'consensus development'/exp OR 'consensus paper':ti,ab OR 'consensus conference':ti,ab OR 'policy statement':ti,ab OR 'policy statements':ti,ab OR 'position statement':ti,ab OR 'position statements':ti,ab**	**52455**
**#36**	**'critical pathways':ti,ab OR 'critical pathway':ti,ab OR 'clinical pathway'/exp**	**11515**
**#35**	**recommendat*:ti,ab**	**446054**
**#34**	**'practice guideline'/exp**	**668880**
**#33**	**guideline*:ti,ab**	**689302**
**#32**	**#28 AND #31**	**3705**
**#31**	**#29 OR #30**	**721781**
**#30**	**nutritional:ti,ab AND (pathway:ti,ab OR pathways:ti,ab OR management:ti,ab OR support:ti,ab OR challenges:ti,ab OR challenge:ti,ab OR deficiency:ti,ab OR deficiencies:ti,ab)**	**73562**
**#29**	**'malnutrition'/exp OR malnutrition:ti,ab OR malnourishment:ti,ab OR malnourishments:ti,ab OR undernutrition:ti,ab OR undernourishment:ti,ab OR malnourished:ti,ab OR feeding:ti,ab OR underfeeding:ti,ab OR nutrition:ti,ab**	**682265**
**#28**	**#15 AND #27**	**123980**
**#27**	**#16 OR #17 OR #18 OR #19 OR #20 OR #21 OR #22 OR #23 OR #24 OR #25 OR #26**	**389274**
**#26**	**'congestive heart failure'/exp OR 'congestive heart failure':ti,ab OR 'cardiac congestive failure':ti,ab OR 'congestive cardiac failure':ti,ab OR 'congestive heart insufficiency':ti,ab**	**146706**
**#25**	**'malformation of heart':ti,ab OR 'heart abnormalities':ti,ab**	**819**
**#24**	**'congenital heart surgeries':ti,ab OR 'congenital heart surgery':ti,ab**	**2613**
**#23**	**'chd':ti,ab**	**44856**
**#22**	**'congenital heart defects':ti,ab OR 'congenital heart defect':ti,ab**	**13144**
**#21**	**'neonatal cardiopathy':ti,ab**	**2**

**#20**	**'heart congenital disease':ti,ab OR 'heart congenital diseases':ti,ab**	**9**
**#19**	**'congenital heart failure':ti,ab**	**104**
**#18**	**'congenital cardiac distress':ti,ab**	**1**
**#17**	**'congenital cardiac disease':ti,ab OR 'congenital cardiac diseases':ti,ab**	**1032**
**#16**	**'congenital heart disease'/exp OR 'congenital heart disease':ti,ab OR 'congenital heart diseases':ti,ab**	**215115**
**#15**	**#1 OR #2 OR #3 OR #4 OR #5 OR #6 OR #7 OR #8 OR #9 OR #10 OR #11 OR #12 OR #13 OR #14**	**4559980**
**#14**	**premature:ti,ab OR prematurity:ti,ab OR preterm:ti,ab OR 'pre term':ti,ab**	**304900**
**#13**	**neonat*:ti,ab**	**397455**
**#12**	**newborn*:ti,ab OR 'new born':ti,ab OR 'new borns':ti,ab OR 'newly born':ti,ab**	**235879**
**#11**	**'infant'/exp OR 'infant':ab,ti OR 'infants':ab,ti OR 'infantile':ab,ti OR 'infancy':ab,ti**	**1462539**
**#10**	**underage*:ti,ab OR 'under age':ti,ab OR 'under aged':ti,ab**	**9111**
**#9**	**youth*:ti,ab**	**113131**
**#8**	**juvenil*:ti,ab**	**114501**
**#7**	**child*:ti,ab OR baby*:ti,ab OR babies:ti,ab**	**2172187**
**#6**	**kid:ti,ab OR kids:ti,ab**	**14000**
**#5**	**girl*:ti,ab**	**226208**
**#4**	**boy:ti,ab OR boys:ti,ab OR boyhood:ti,ab**	**218386**
**#3**	**pediatric*:ti,ab OR peadiatric*:ti,ab OR paediatric*:ti,ab**	**670453**
**#2**	**'pediatrics'/exp**	**132430**
**#1**	**'child'/exp**	**3250571**

Table S2. List of additional scientific societies.

**Name of the scientific societies**	**Country**
The National Institute for Health and Care Excellence (NICE)	United Kingdom
The Canadian Agency for Drugs & Technologies in Health (CADTH)	Canada
Scottish Intercollegiate Guidelines Network (SIGN)	Scotland
European Society of Cardiology	
American Coolege of Cardiology	USA
Association for European Paediatric and Congenital Cardiology	
American Society for Parenteral and Enteral Nutrition	USA

WHO	
he Agency for Healthcare Research and Quality's (AHRQ)	USA
National Health and Medical Research Council (NHMRC)	Australia
ECRI Institute	USA
The Belgian Health Care Knowledge Centre (KCE)	Belgium
Polskie Towarzystwo Kardiologiczne	Poland
Polskie Towarzystwo Pediatryczne	Poland
Royal College of Paediatrics and Child Health	United Kingdom
The European Society of Pediatric and Neonatal Intensive Care (ESPNIC)	Europe

Table S3. Detailed summary of inclusion and exclusion criteria according to PICAR Framework.

**Inclusion criteria**	
**P**opulation and clinical indications, and conditions	- Children (0-18 years) with CHD and malnutrition
**I**nterventions	Any nutritional intervention
**C**omparators	- No comparator<br>- Standard nutritional protocol for the healthy pediatric population according to WHO, AAP, EFSA or ESPGHAN<br>- Standard strategies in the treatment of malnutrition in children according to local guidelines
**A**ttributes of CPGs	- Language: English<br>- Version: Only the latest version is of interest<br>- Development process: Evidence- and/or consensus-based<br>- System of rating evidence: any<br>- Scope: CPGs must be primarily focused on the nutritional recommendations in CHD<br>- Recommendations: CPGs will only be included if they report one or more eligible recommendations of interest
**R**ecommendation characteristics	- Interventions: Recommendations must explicitly discuss the intervention of interest

	- Comparators: Recommendations are not required to compare an intervention of interest to any other nutritional protocol. If such a comparison is made, the comparator must also meet specific eligibility criteria (see *Comparators*)<br>- Duration of treatment: Recommendations on the duration of nutritional intervention are of interest<br>- Levels of confidence: Each recommendation should be accompanied by an explicit level of confidence<br>- Locating recommendations: within CPG text, tables, algorithms, and/or decision paths
**Exclusion criteria**	
- other language of publication than English	
- no system of rating evidence used by the authors	
- other population than children with CHD	

Abbreviations: WHO – World Health Organisation; AAP – Amedican Academy of Pediatrics; EFSA – European Food Safety Authority; ESPGHAN – European Society for Paediatric Gastroenterology, Hepatology and Nutrition; CPGs – Clinical Practice Guidelines; CHD – Congenital Heart Disease.

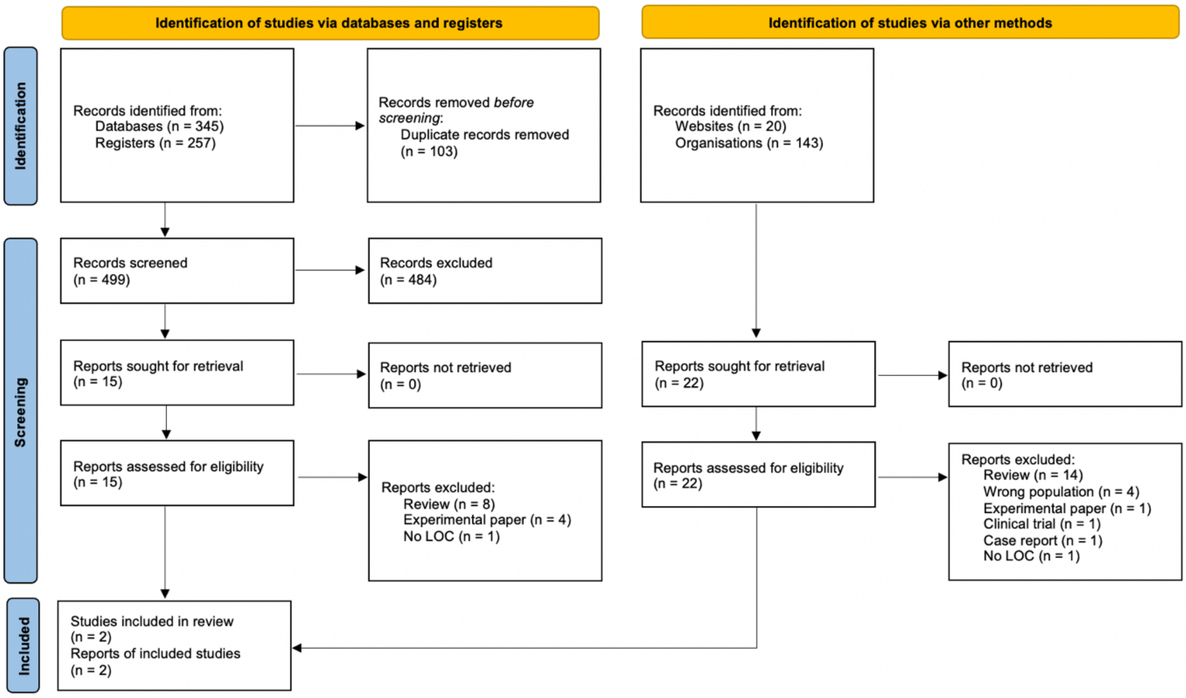

Figure S1. Summary of evidence search and selection using PRISMA 2020 flow diagram for new systematic reviews.
